# Time course of redox potential and antioxidant capacity in patients undergoing cardiac surgery

**DOI:** 10.1186/cc14104

**Published:** 2015-03-16

**Authors:** C Stoppe, G Schaelte, S Kraemer, C Benstoem, D Bar-Or, A Goetzenich

**Affiliations:** 1 RWTH Aachen University, Aachen, Germany; 2 RWTH Aachen University, University Hospital, Aachen, Germany; 3 Swedish Medical Center, Trauma Research, Engelwood, CO, USA

## Introduction

Cardiac surgery regularly provokes inflammation and oxidative stress which contribute to the development of organ failure and mortality of patients. While the assessment of single markers does not reflect a comprehensive investigation of redox status, the measurement of oxidation-reduction potential (ORP) provides a reliable measure to assess the balance between total prooxidant and antioxidant balance in the blood. The aim of the present study was to investigate the overall redox potential in patients undergoing cardiac surgery.

## Methods

This is a prospective observational study in patients scheduled for elective cardiac surgery. Serum samples were drawn prior to surgery, after connection to cardiopulmonary bypass (ischemia), after opening of cross-clamp (reperfusion) and after termination of surgery.

The redox status of patients was measured using the bedside point of care RedoxSYS Diagnostic System™ (Luoxis, USA). Simultaneously the antioxidant capacity in serum samples were calculated in all perioperatively obtained serum samples.

## Results

**Figure 1:**
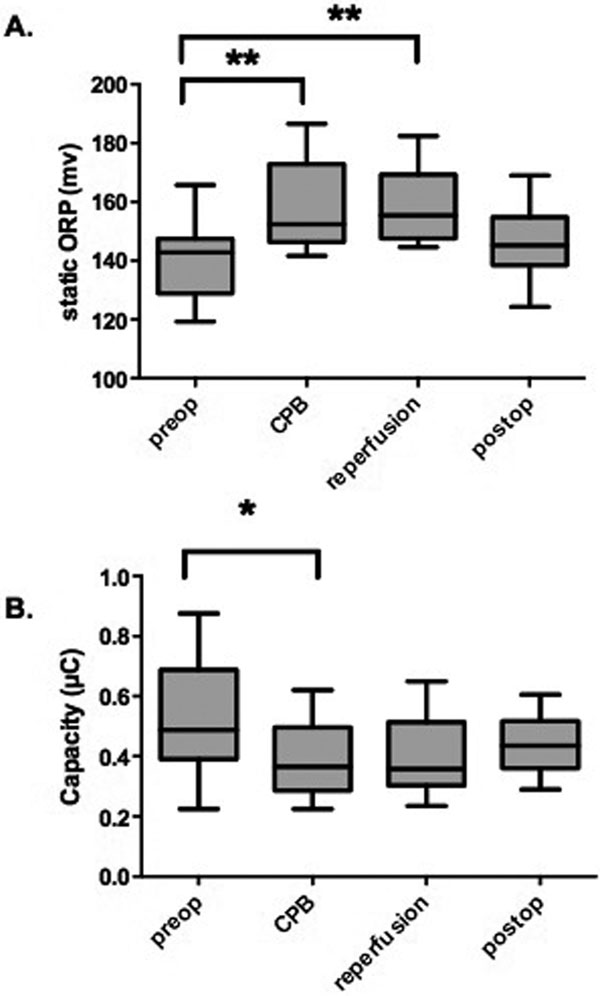
**(A), (B) Perioperative time course of oxidative stress and antioxidant capacity**.

All patients' sera (*n *= 17) demonstrated a significant increase of ORP upon start of myocardial ischemia (141.0 ± 4.8 mV vs. 157.9 ± 4.9 mV; *P *= 0.002) and compared with reperfusion (141.0 ± 4.8 mV vs. 158.6 ± 4.9mV; *P *< 0.001, Figure 1A). In parallel, the antioxidant capacity significantly decreased during surgery (0.505 ± 0.190 μC vs. 0.384 ± 0.120 μC; *P *= 0.022) corresponding to the increase of oxidative stress (Figure 1B).

## Conclusion

This preliminary study is the first to highlight the time course of overall redox potential and antioxidant capacity in cardiac surgery patients. Further studies are underway to evaluate the clinical significance on outcome in cardiac surgery patients.

